# Effect of orthodontic extraction of mandibular premolars on third molar angulation after treatment with fixed appliances: A cross-sectional study

**DOI:** 10.1007/s00056-023-00465-3

**Published:** 2023-03-31

**Authors:** Tamara Di Giovanni, Theodosia Vogiatzi, Vasiliki Koretsi, Tanya Walsh, Nick Silikas, Spyridon N. Papageorgiou

**Affiliations:** 1 Clinic of Orthodontics and Pediatric Dentistry, Center of Dental Medicine, University of Zurich, Plattenstr. 11, 8032 Zurich, Switzerland; 2 Division of Dentistry, School of Medical Sciences, Faculty of Biology, Medicine and Health, University of Manchester, Manchester, UK

**Keywords:** Premolar extraction, Third molars, Impacted tooth, Orthodontic treatment, Retrospective cross-sectional study, Extraktion von Prämolaren, Dritte Molaren, Impaktierter Zahn, Kieferorthopädische Behandlung, Retrospektive Querschnittsstudie

## Abstract

**Purpose:**

Orthodontic treatment involving premolar extractions might improve the angulation of lower third molars, which are the teeth most often impacted. This study analyzes the impact of first/second lower premolar extraction during orthodontic therapy on the angulation of mandibular third molars.

**Methods:**

A total of 120 patients treated non-extraction (*n* = 40), with extraction of first (*n* = 40), or second lower premolars (*n* = 40) were included. The mesiodistal angulation of lower third molars relative to the adjacent tooth and their developmental stage were evaluated from posttreatment orthopantomograms. Between-group differences were statistically evaluated at a significance level of 0.05.

**Results:**

The orthopantomograms of 120 patients (51% female) with a median age of 15.2 years at the time of debonding were evaluated after a mean treatment duration time of 2.9 years. No difference (*P* > 0.05) was seen between the average angulation of the lower third molars of the right (mean = 24.4°, standard deviation [SD] 13.6°) and the left side (mean = 23.6°, SD 14.1°). No differences in the angulation of the lower third molar were found between the non-extraction and extraction groups for the right (*P* = 0.44) or the left side (*P* = 0.22). Likewise, no differences were found when comparing the first and second premolars for the right (*P* = 0.26) or the left side (*P* = 0.10). Premolar extraction was associated with an advanced root development stage of the right third molar (odds ratio 7.1; 95% confidence interval 1.1–48.1; *P* = 0.04), with no differences between extraction of the first or second premolar (*P* = 0.10).

**Conclusion:**

Orthodontic treatment involving premolars extractions might be associated with a small acceleration in root development, but not with the angulation, of lower third molars.

**Supplementary Information:**

The online version of this article (10.1007/s00056-023-00465-3) contains supplementary material, which is available to authorized users.

## Abstract (de)

**Zielsetzung:**

Eine kieferorthopädische Behandlung mit Extraktion von Prämolaren könnte die Angulation der unteren dritten Molaren verbessern, die am häufigsten impaktiert sind. In dieser Studie wird der Einfluss der Extraktion erster/zweiter unterer Prämolaren während einer kieferorthopädischen Therapie auf die Angulation der dritten Molaren im Unterkiefer untersucht.

**Methoden:**

Insgesamt wurden 120 Patienten eingeschlossen, die ohne Extraktion (*n* = 40), mit Extraktion der ersten (*n* = 40) bzw. der zweiten unteren Prämolaren (*n* = 40) behandelt wurden. Die mesiodistale Angulation der unteren dritten Molaren in Relation zum Nachbarzahn und das Entwicklungsstadium wurden anhand von Orthopantomogrammen nach der Behandlung bewertet. Die Unterschiede zwischen den Gruppen wurden statistisch auf einem Signifikanzniveau von 0,05 ausgewertet.

**Ergebnisse:**

Die Orthopantomogramme von 120 Patienten (51% weiblich) mit einem mittleren Alter von 15,2 Jahren zum Zeitpunkt des Debonding wurden nach einer mittleren Behandlungsdauer von 2,9 Jahren ausgewertet. Es wurde kein Unterschied (*p* = 0,05) zwischen der durchschnittlichen Angulation der unteren dritten Molaren der rechten (Mittelwert = 24,4°, Standardabweichung [SD] 13,6°) und der linken Seite (Mittelwert = 23,6°, SD 14,1°) festgestellt. Es wurden keine Unterschiede in der Angulation des unteren dritten Molaren zwischen der Nicht-Extraktions- und der Extraktionsgruppe für die rechte (*p* = 0,44) bzw. die linke Seite (*p* = 0,22) festgestellt. Ebenso wurden keine Unterschiede beim Vergleich der ersten und zweiten Prämolaren auf der rechten (*p* = 0,26) bzw. der linken Seite (*p* = 0,10) festgestellt. Die Extraktion von Prämolaren war mit einem fortgeschrittenen Wurzelentwicklungsstadium des rechten dritten Molaren verbunden (Odds Ratio 7,1; 95%-Konfidenzintervall 1,1–48,1; *p* = 0,04), ohne Unterschiede zwischen der Extraktion des ersten oder des zweiten Prämolaren (*p* = 0,10).

**Schlussfolgerung:**

Eine kieferorthopädische Therapie mit Extraktion von Prämolaren könnte mit einer leichten Beschleunigung der Wurzelentwicklung, nicht aber mit der Angulation der unteren dritten Molaren assoziiert sein.

## Introduction

### Rationale

Third molars are the most frequently impacted permanent teeth and account for 98% of all tooth impactions, with a worldwide prevalence of 24% [7]. In addition, mandibular third molars are significantly more often impacted (25.4%) than the maxillary molars (14.2%) and third molar impaction seems to be affected by geographic region, where Asian and Middle Eastern populations seem to be more affected [7]. Impaction of the third molars might be associated with several complications, including infection, cysts, caries, or root resorption of the adjacent second molars [21].

Chronologically, the buds of the lower third molars start forming at the age of 8–9 years, are usually angulated mesially during their calcification, and emerge into the oral cavity at around 17–21 years of age, even though the third molar’s roots are not fully formed until the age of 18–25 years [19]. During their eruption process a continuous angulation change and various pre-eruptive rotational movements occur, making their eruption path often irregular, with highly variability in their formation, calcification, and final eruption position [14]. The third molars’ eruption fate is thought to be affected by many factors [41], including among others tooth morphology, mesiodistal width, unfavorable uprighting, retardation in third molars’ maturation, vertical condylar growth direction, and reduced mandibular length [8]. According to Richardson, the initial angulation of the lower third molars may also influence their subsequent eruption [29]. A twin study, looking at genetic, epigenetic, and environmental factors [41], showed that dental maturation and development is not identical and thus not exclusively gene related, with specific and common environmental factors contributing about one third to the total variation. The study determined that epigenetics specify more the dental traits, whereas mandibular length is significantly influenced by genes [40].

However, a deficiency of retromolar space is assumed to be the main reason for third molar impaction [5, 8, 24, 29, 38, 41]. Anterior resorption at the mandibular ramus and the pattern of eruption of the mandibular dentition play a major part in the formation of retromolar space [3, 29, 38]. In addition to biological factors, retromolar space can be affected by orthodontic treatment including mesialization of the posterior teeth of the dental arch and might be associated with improved position of the third molars [8]. Particularly orthodontic treatment involving extraction of lower premolars and subsequent space closure is assumed to be associated with an increased retromolar space, subsequent increased uprighting and reduced impaction of the mandibular third molars [12]. A recent systematic review [21] reported that the extraction-based orthodontic treatment might be associated with improved third molar angulation by 10–18°, even though the evidence was limited and no clear differentiation was made according to which premolar was extracted.

### Objectives

The aim of the present study was to investigate the effect of comprehensive orthodontic treatment with and without extraction of lower premolars on the angulation of lower permanent third molars. The primary null hypothesis was that there is no difference in the angulation of lower permanent third molars between post-adolescent patients treated orthodontically with fixed appliances combined with extraction of lower first or second premolars and patients treated without extractions. As a secondary objective, the study aimed to identify the influence of premolar extractions on the root mineralization stage of the third molars.

## Materials and methods

Patients for this cross-sectional study were identified from the archive of treated patients in the Clinic of Orthodontics and Pediatric Dentistry at the University of Zurich. All patients had been treated orthodontically in the postgraduate clinic by postgraduate orthodontic residents under the supervision of experienced clinical instructors during the years 2005–2020. Data extraction and measurements were performed from de-identified files having the patients consent for the use of their data in research and the appropriate ethical approval (BASEC 202202225). This report follows the Strengthening the Reporting of Observational Studies in Epidemiology (STROBE) statement [44].

Eligible for this study were patients of any age, gender, or ethnicity, receiving comprehensive orthodontic treatment with fixed appliances in both jaws for any kind of malocclusion, with at least one lower permanent third molar visible in the posttreatment orthopantomogram (OPG), and treated with (i) bilateral extraction of lower first premolars, (ii) bilateral extraction of lower second premolars, or (iii) without premolar extractions. Patients had a full complement of teeth including the third molar(s), no previous orthodontic treatment, no dentofacial deformities or clefts, and a complete set of pretreatment and posttreatment records (patient file, extraoral/intraoral photographs, dental cast models, OPG, and lateral cephalogram). Patient eligibility was determined irrespective of any potential confounders to the eruption rate of third molars like patient age, posterior space availability, third molar root development stage, vertical position, or angulation.

Among studies comparing premolar extraction with non-extraction treatment groups, the sample size in the exposed (extraction) group ranged from 10 to 134 patients (median of 25 patients per group) [2, 8, 11, 12, 15, 17, 25, 27, 30, 32, 34, 38, 39]. A naïve sample size calculation was undertaken taking the expected third molar angulation for the non-extraction group from the largest study (mean 24.21°; standard deviation [SD] 3.7°) [11]. Assuming a 20% change of third molar inclination as a clinically relevant effect from extraction treatment, same SD, alpha of 5%, beta of 10% (power of 90%), a total of 14 patients per extraction/non-extraction group would be needed. But as this study aimed to also assess differences according to the extraction of first/second premolars and according the third molars’ developmental stage, an increased sample size was sought. Therefore, the sample size was arbitrarily chosen at 40 patients per group: 40 patients treated with extraction of the two lower first premolars, 40 patients treated with extraction of the two lower second premolars and 40 patients treated without any premolar extractions (total of 120 patients).

The first 120 eligible patients were selected consecutively according to their archive number, starting from the most recent cases, and moving to the past, until the desired sample was collected. All OPGs evaluated for this study had been taken as part of the standard documentation after removal of the fixed appliances to check for root resorption in-house (CRANEX D, Sordex Dental Imaging, Germany; 73KV, 10 mA).

All patients had been treated with standard Edgewise appliances (Mini Twin Diamond; Ormco, Orange, CA, USA), conventionally ligated, and with an 0.018-inch slot. Treatment mechanics (including space closure and torque retention) were left to the discretion of the clinical instructors supervising the treatment, but space closure mostly included closing loops on slot-filling rectangular wires and no skeletal anchorage devices were used.

**Fig. 1:**
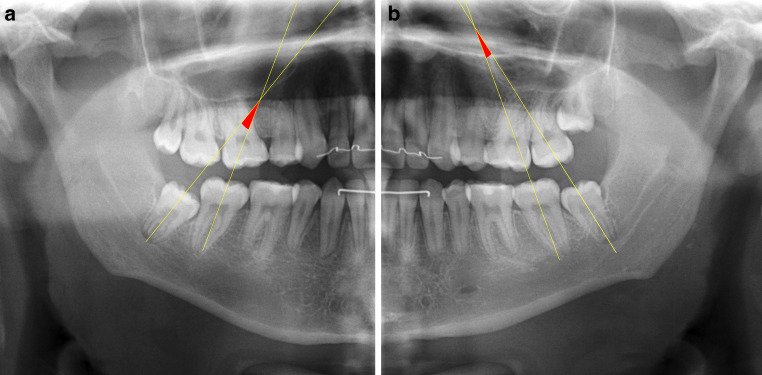
Lines used to measure the third molar angulation on the orthopantomograms. Measured angulations of +16.19° for the patient’s right third molar (**a**) and +8.65° for the patient’s left third molar (**b**) Zur Messung der Angulation des dritten Molaren auf den Orthopantomogrammen verwendete Linien. Gemessene Winkel von +16,19° für den rechten dritten Molaren des Patienten (**a**) und +8,65° für den linken dritten Molaren des Patienten (**b**)

The 120 posttreatment OPGs included were assessed in a blinded manner (the assessor did not know the group to which the OPG belonged to) with Digimizer image analysis software (MedCalc Software, Mariakerke, Belgium). For the measurement of the angulation of the third molar, the long axis of second and third molars were drawn, aiming for the middle of the molars’ occlusal crown area and the root furcation points (Fig. 1). The angulation of both the right and left lower third molar were measured if both molars existed on the posttreatment OPG. In cases of unilateral agenesis of the lower third molar, only the remaining tooth was measured. All measurements were performed by one dentist (TDG) and one maxillofacial radiologist (TV). After a period of 4 weeks, all measurements were repeated.

Dental development stage was evaluated using the method of Demirjian et al. [9], which is based on eight stages of tooth formation. The first four stages (A–D) show crown formation from the beginning of cusp calcification to completed crown, and the second four (E–H) show root formation from initial radicular bifurcation to apical closing. These were evaluated both as raw stages, as well as categories of crown formation (stages A–D), mid-root formation (stages E–F), and root completion (stages G–H).

Descriptive statistics were calculated, consisting of absolute/relative frequencies for the categorical variables. For continuous variables, data were checked for normality through visual inspection of histograms and formally with the Shapiro–Wilk test. For normally distributed data, means and SDs were calculated. For skewed data, medians and interquartile ranges (IQR) were calculated. Differences between non-extraction/extraction groups were assessed with Fisher’s exact tests for categorical data, with independent samples t‑test or one-way analysis of variance for normally distributed continuous data (after checking assumptions), and with Mann–Whitney or Kruskal–Wallis tests for skewed continuous data. General linear models were constructed to assess any difference between angulation of the right and left molars, taking within-patient clustering into account with robust standard errors. Logistic regression was used for the outcome of a third molar having roots developed more than 50% [9] constructing either a crude model or one adjusting for age at debonding and reporting odds ratios (OR) with their 95% confidence intervals (CI) using an overall Wald test for predictors with > 2 levels. Repeatability and agreement of the measurements were assessed with the concordance correlation coefficient (CCCs) [20] and the Bland–Altman method [6]. Alpha was set at a two-sided *P* < 0.05, all analyses were done in Stata SE 14.2 (StataCorp, College Station, TX, USA), and the data set was openly provided [10].

## Results

### Patient demographics

A total of 120 patients satisfied all eligibility criteria and were included, 59 (49%) were male and the median age of the sample was 15.2 years at the time of debonding (IQR 13.9–16.9 years; Table 1). Of these patients, 40 were treated without lower premolar extractions and 80 were treated with extraction of either the first (*n* = 40) or the second lower premolar (*n* = 40), with a mean treatment duration of 2.9 years (SD 1.0 year). Both right and left lower third molars were visible in the posttreatment OPGs of 118 patients (98%), while in 2 patients only the right lower third molar existed in the posttreatment OPG (2%).

**Table 1:** Demographics and results of non-extraction or extraction group Demografische Angaben und Ergebnisse der Nicht-Extraktion- und der Extraktion-Gruppe

Variable	Measure	Non-extraction (*n* = 40)	Premolar extraction (*n* = 80)	Total (*n* = 120)	*P*	Mean difference (95% CI)
Sex	Female, *n* (%)	21 (53%)	40 (50%)	61 (51%)	0.80^a^	–
	Male, *n* (%)	19 (48%)	40 (50%)	59 (49%)		
Age at debond	Median (IQR)	15.8 (13.8, 17.2)	15.1 (14.0, 16.6)	15.2 (13.9, 16.9)	0.49^b^	–
Extraction	1^st^ premolar, *n* (%)	–	40 (50%)	40 (50%)	NT	–
	2^nd^ premolar, *n* (%)	–	40 (50%)	40 (50%)		
Treatment duration (years)	Mean (SD)	2.9 (1.1)	2.9 (0.9)	2.9 (1.0)	0.97^c^	0.01 (−0.4 to 0.4)
Third molars	48 & 38, *n* (%)	40 (100%)	78 (98%)	118 (98%)	0.55^d^	–
	48, *n* (%)	0 (0%)	2 (3%)	2 (2%)		
Angulation 48	Mean (SD)	25.8 (14.3)	23.7 (13.3)	24.4 (13.6)	0.44^c^	2.1 (−3.2 to 7.3)
Angulation 38	Mean (SD)	24.6 (13.1)	23.1 (14.7)	23.6 (14.1)	0.59^c^	1.5 (−3.9 to 6.9)
Difference 48–38	Mean (SD)	1.2 (13.2)	0.7 (10.2)	0.9 (11.2)	0.84^c^	−1.8 (−6.5 to 2.9)

^a^ χ^2^ test

^b^ Mann–Whitney test

^c^ independent t‑test

^d^ Fisher exact test

*IQR* interquartile range; *NT* not tested; *SD* standard deviation, *CI* confidence interval

### Repeated measurements

Repeatability and agreement were found to be excellent for both interexaminer comparisons (CCCs 0.93–0.94; average differences 0.63–1.41°) and for the repeated intraexaminer comparisons (CCCs 0.98–0.99; average differences 0.31–0.70°; Supplementary Table 1). However, relatively broad limits of agreement were seen for both interexaminer and intraexaminer comparisons, which indicate that some variability existed in the measured angulation of the lower third molars.

### Extraction versus non-extraction treatment

**Fig. 2:**
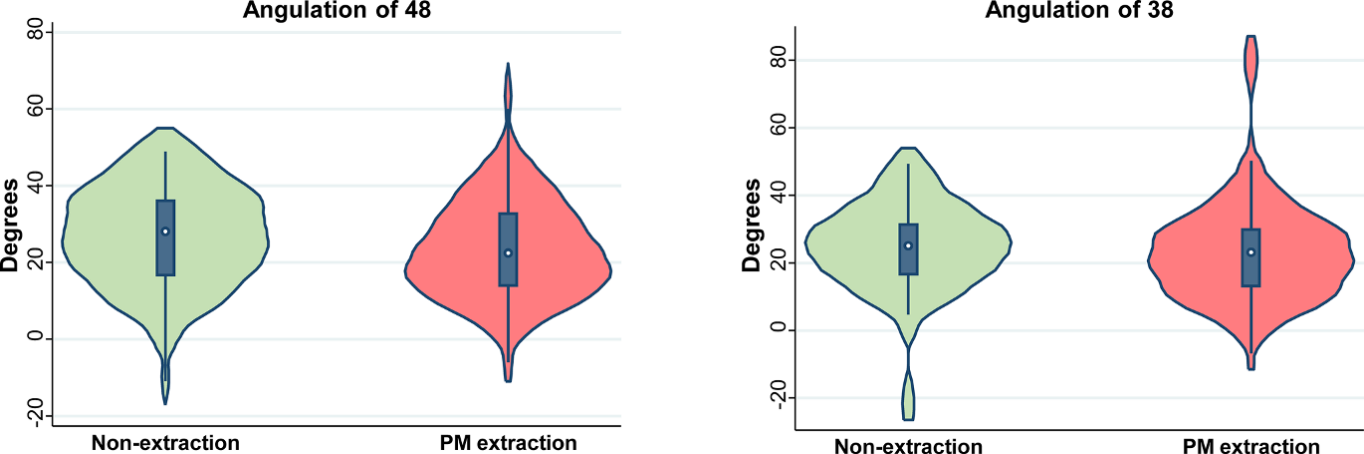
Violin plots for third molar angulation in the non-extraction and extraction groups. *PM* premolar Violin-Plots für die Angulation der dritten Molaren in der Nicht-Extraktion- und in der Extraktion-Gruppe. *PM* Prämolar

No statistically significant differences in patient gender, age at time of debonding, treatment duration, or number of assessed third molars existed between the extraction and non-extraction groups (*P* > 0.05 in all instances; Table 1). The mean angulation of the lower right third molar in relation to the adjacent second molar was 24.4° (SD 13.6°) with no statistically significant difference between the non-extraction group (mean 25.8°; SD 14.3°) and the premolars extraction group (mean 23.7°; SD 13.3°; Fig. 2), with a difference in means of 2.1° (95% CI −3.2 to 7.3; *P* = 0.44). The mean angulation of the lower left third molar in relation to the adjacent second molar was 23.6° (SD 14.1°) for all groups with no significant differences between the non-extraction group (mean 24.6°; SD 13.1°) and the premolars extraction group (mean 23.1°; SD 14.7°; difference 1.5°; 95% CI −3.9 to 6.9; *P* = 0.59). In addition, no differences were seen between the average angulation difference between the right and left lower third molars among all patients.

### Differences between extraction of first or second premolars

**Fig. 3:**
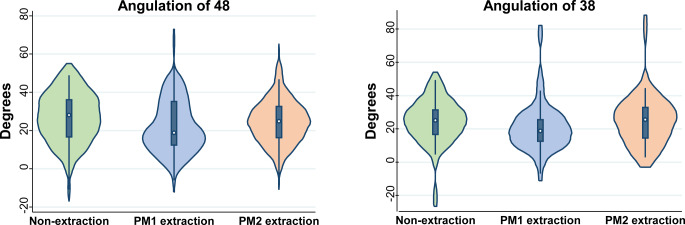
Violin plots for third molar angulation in the non-extraction and extraction groups, according to the premolar extracted. *PM* premolar Violin-Plots für die Angulation der dritten Molaren in der Nicht-Extraktion- und in der Extraktion-Gruppe, je nach extrahiertem Prämolaren. *PM* Prämolar

For the analysis of third molar angulation comparing non-extraction versus first premolar extraction or second premolar extraction, data were positively skewed and therefore nonparametric statistics were used. When comparing non-extraction patients with patients with extractions of first premolars and patients with extractions of second premolars, no significant differences were found with regard to patient gender, age at time of debonding, treatment duration, or number of assessed third molars (*P* > 0.05 in all instances; Table 2). The median angulation of the lower right third molar in relation to the adjacent second molar was 24.2° (IQR 15.2–33.0°) with no significant difference between the non-extraction group (median 28.2°; IQR 16.7–36.1°), the first premolars extraction group (median 19.0°; IQR 12.5–35.2°), and the second premolars extraction group (median 24.9°; IQR 16.4–32.6°; Fig. 3; *P* = 0.26). Likewise, small variation was seen in the median angulation of the lower left third molar in relation to the adjacent second molar when comparing the non-extraction group (median 25.1°; IQR 16.7–31.4°) with the first premolars extraction group (median 18.9°; IQR 12.6–25.6°), and the second premolars extraction group (median 25.7°; IQR 14.5–32.9°), which was again not statistically significant (*P* = 0.10). Finally, the difference between the right and left third molars did not reach statistical significance between the non-extraction group, the first premolars extraction group, and the second premolars extraction group (*P* = 0.72). In conclusion, the null hypothesis could not be rejected for either tooth 38 or 48 and according to which premolar was extracted (Table 2).

**Table 2:** Demographics and results of non-extraction or extraction group, according to the premolar extracted Demografische Angaben und Ergebnisse der Nicht-Extraktion- und der Extraktion-Gruppe, je nach extrahiertem Prämolaren

Variable	Measure	Non-extraction (*n* = 40)	Extraction 1st premolar (*n* = 40)	Extraction 2nd premolar (*n* = 40)	Total (*n* = 120)	*P*
Sex	Female, *n* (%)	21 (53%)	20 (50%)	20 (50%)	61 (51%)	0.97^a^
	Male, *n* (%)	19 (48%)	20 (50%)	20 (50%)	59 (49%)	
Age at debond	Median (IQR)	15.8 (13.8, 17.2)	14.6 (13.7, 16.5)	15.3 (14.5, 16.7)	15.2 (13.9, 16.9)	0.42^b^
Treatment duration	Mean (SD)	2.9 (1.1)	2.8 (0.9)	2.9 (1.0)	2.9 (1.0)	0.96^c^
Third molars	48 & 38, *n* (%)	40 (100%)	38 (95%)	40 (100%)	118 (98%)	0.33^d^
	48, *n* (%)	0 (0%)	2 (5%)	0 (0%)	2 (2%)	
Angulation 48	Median (IQR)	28.2 (16.7, 36.1)	19.0 (12.5, 35.2)	24.9 (16.4, 32.6)	24.2 (15.2, 33.0)	0.26^b^
Angulation 38	Median (IQR)	25.1 (16.7, 31.4)	18.9 (12.6, 25.6)	25.7 (14.5, 32.9)	23.8 (14.1, 31.1)	0.10^b^
Difference 48–38	Mean (SD)	1.2 (13.2)	1.8 (10.0)	−0.3 (10.4)	0.9 (11.2)	0.72^c^

^a^ χ^2^ test

^b^Kruskal–Wallis test

^c^One-way analysis of variance

^d^Fisher exact test, with extension from Mehta and Patel [22]

*IQR* interquartile range; *SD* standard deviation

### Dental development stage differences

At the time of the posttreatment OPGs, all lower right third molars were allocated to the Demirjian stages B–H (Table 3), while no clear Demirjian staging could be performed for tooth 48 in 3 non-extraction patients and for tooth 38 in 6 patients (3 in the extraction and 3 in the non-extraction group). Differences between the developmental stage and extraction groups were undertaken using Fisher’s exact test extended to multiple rows and columns [22]. For the lower right third molars, 44% (*n* = 51) of them were in the crown development stage, 40% (*n* = 47) of them had up to half of their roots developed, and 16% (*n* = 19) of them had more progressed/complete root formation. No statistically significant differences between extraction and non-extraction patients were seen either using the separate stages (*P* = 0.39) or the developmental categories (*P* = 0.15). The lower left third molars were allocated to the Demirjian stages C–H [9], with 45% (*n* = 51) of them being in the crown development stage, 41% (*n* = 47) of them having up to half of their roots developed, and 14% (*n* = 16) having more progressed/complete root formation. Similarly, no significant differences were seen between extraction and non-extraction patients using either the separate stages (*P* = 0.25) or the developmental categories (*P* = 0.32).

**Table 3:** Dental development stage differences between non-extraction and extraction groups^b^ Unterschiede im Zahnentwicklungsstadium zwischen den Gruppen: Nicht-Extraktion und Extraktion^b^

Tooth	Demirjian stage	Non-extraction (*n* = 40)	Premolar extraction (*n* = 80)	Total (*n* = 120)	*P*^a^
48—raw	A, *n* (%)	0 (0%)	0 (0%)	0 (0%)	0.39
	B, *n* (%)	0 (0%)	1 (1%)	1 (1%)	
	C, *n* (%)	4 (11%)	7 (9%)	11 (9%)	
	D, *n* (%)	11 (30%)	28 (35%)	39 (33%)	
	E, *n* (%)	15 (41%)	18 (23%)	33 (28%)	
	F, *n* (%)	4 (11%)	10 (13%)	14 (12%)	
	G, *n* (%)	3 (8%)	11 (14%)	14 (12%)	
	H, *n* (%)	0 (0%)	5 (6%)	5 (4%)	
48—category	A–D, *n* (%)	15 (41%)	36 (45%)	51 (44%)	0.15
	E–F, *n* (%)	19 (51%)	28 (35%)	47 (40%)	
	G–H, *n* (%)	3 (8%)	16 (20%)	19 (16%)	
38—raw	A, *n* (%)	0 (0%)	0 (0%)	0 (0%)	0.25
	B, *n* (%)	0 (0%)	0 (0%)	0 (0%)	
	C, *n* (%)	4 (11%)	7 (9%)	11 (10%)	
	D, *n* (%)	10 (27%)	30 (39%)	40 (35%)	
	E, *n* (%)	16 (43%)	17 (22%)	33 (29%)	
	F, *n* (%)	3 (8%)	11 (14%)	14 (12%)	
	G, *n* (%)	4 (11%)	9 (12%)	13 (11%)	
	H, *n* (%)	0 (0%)	3 (4%)	3 (3%)	
38—category	A–D, *n* (%)	14 (38%)	37 (48%)	51 (45%)	0.32
	E–F, *n* (%)	19 (51%)	28 (36%)	47 (41%)	
	G–H, *n* (%)	4 (11%)	12 (15%)	16 (14%)	

^a^ Fisher exact test, with extension from Mehta and Patel [22]

^b^Stage categorization was unclear for tooth 48 in 3 non-extraction patients and for tooth 38 in 6 patients (3 per group) and was omitted.

Differences were observed in the separate staging of the lower right and left third molar between the non-extraction, first premolar extraction, and second premolar extraction groups. Due to the sparseness of the data (many cells with zero observations), we did not undertake formal statistical analysis. However, when the distribution between the three patient groups was analyzed with regard to the categorized stages, there was no evidence of a difference for the right molar (*P* = 0.17) or the left molar (*P* = 0.43; Table 4).

**Table 4:** Dental development stage differences between non-extraction and extraction groups, according to the premolar extracted^b^ Unterschiede im Zahnentwicklungsstadium zwischen den Gruppen Nicht-Extraktion und Extraktion, je nach extrahiertem Prämolaren^b^

Tooth	Demirjian stage	Non-extraction (*n* = 40)	Extraction 1st premolar (*n* = 40)	Extraction 2nd premolar (*n* = 40)	Total (*n* = 120)	*P*^a^
48—raw	A, *n* (%)	0 (0%)	0 (0%)	0 (0%)	0 (0%)	NC
	B, *n* (%)	0 (0%)	0 (0%)	1 (3%)	1 (1%)	
	C, *n* (%)	4 (11%)	1 (3%)	6 (15%)	11 (9%)	
	D, *n* (%)	11 (30%)	18 (45%)	10 (25%)	39 (33%)	
	E, *n* (%)	15 (41%)	6 (15%)	12 (30%)	33 (28%)	
	F, *n* (%)	4 (11%)	5 (13%)	5 (13%)	14 (12%)	
	G, *n* (%)	3 (8%)	5 (13%)	6 (15%)	14 (12%)	
	H, *n* (%)	0 (0%)	5 (13%)	0 (0%)	5 (4%)	
48—category	A–D, *n* (%)	15 (41%)	19 (48%)	17 (43%)	51 (44%)	0.17
	E–F, *n* (%)	19 (51%)	11 (28%)	17 (43%)	47 (40%)	
	G–H, *n* (%)	3 (8%)	10 (25%)	6 (15%)	19 (16%)	
38—raw	A, *n* (%)	0 (0%)	0 (0%)	0 (0%)	0 (0%)	NC
	B, *n* (%)	0 (0%)	0 (0%)	0 (0%)	0 (0%)	
	C, *n* (%)	4 (11%)	1 (3%)	6 (15%)	11 (10%)	
	D, *n* (%)	10 (27%)	18 (49%)	12 (30%)	40 (35%)	
	E, *n* (%)	16 (43%)	6 (16%)	11 (28%)	33 (29%)	
	F, *n* (%)	3 (8%)	5 (14%)	6 (15%)	14 (12%)	
	G, *n* (%)	4 (11%)	4 (11%)	5 (13%)	13 (11%)	
	H, *n* (%)	0 (0%)	3 (8%)	0 (0%)	3 (3%)	
38—category	A–D, *n* (%)	14 (38%)	19 (51%)	18 (45%)	51 (45%)	0.43
	E–F, *n* (%)	19 (51%)	11 (30%)	17 (43%)	47 (41%)	
	G–H, *n* (%)	4 (11%)	7 (19%)	5 (13%)	16 (14%)	

^a^ Fisher exact test, with extension from Mehta and Patel [22]

*NC* not calculated due to sparse data

^b^Stage categorization was unclear for tooth 48 in 3 non-extraction patients and for tooth 38 in 6 patients (3 non-extraction and 3 in the second premolar extraction group) and was omitted

The adjusted for age logistic regression analyses indicated that premolar extractions were associated with increased odds of the lower right third molar having advanced root formation (OR 7.12, 95% CI 1.05–48.17; *P* = 0.04; Table 5). Analysis according to the premolar extracted indicated that the effect was not statistically significant between the different premolar extraction groups and non-extraction groups (χ^2^ = 4.64; *P* = 0.10). The adjusted for age logistic regression analyses indicated no statistically significant effect of premolar extractions on the odds of the lower left third molar having advanced root formation (OR = 2.84; 95% CI 0.56–14.53; *P* = 0.21; Table 5) and similarly no difference according to whether the first or second premolar was extracted (χ^2^ = 2.67; *P* = 0.26).

**Table 5:** Logistic regression on the outcome of third molar having at least half of root mineralization completed (Demirjian stages G–H) Logistische Regression zum Ergebnis des dritten Molaren mit mindestens zur Hälfte abgeschlossener Wurzelmineralisierung (Demirjian-Stadien G-H)

		Tooth 48				Tooth 38			
		Crude model		Adjusted model		Crude model		Adjusted model	
Variable	Category	OR (95% CI)	*P*	OR (95% CI)	*P*	OR (95% CI)	*P*	OR (95% CI)	*P*
Extractions	No	Reference		Reference		Reference		Reference	
	PMs	2.83 (0.77, 10.41)	0.12	7.12 (1.05, 48.13)	0.04	1.52 (0.46, 5.09)	0.49	2.84 (0.56, 14.53)	0.21
Extractions	No	Reference		Reference		Reference		Reference	
	PM1	3.78 (0.95, 15.02)	0.15^a^	13.52 (1.22, 149.67)	0.10^a^	1.93 (0.51, 7.24)	0.57^a^	5.87 (0.70, 49.26)	0.26^a^
	PM2	2.00 (0.46, 8.66)	–	5.56 (0.73, 42.51)	–	1.18 (0.29, 4.77)	–	2.04 (0.34, 12.41)	–

*CI* confidence interval, *OR* odds ratio, *PM* premolar

^a^Overall Wald test

## Discussion

The findings of this retrospective cross-sectional study indicate that no statistically significant difference in the lower third molar angulation was observed between the premolar extraction and non-extraction groups.

Existing studies in the published literature report significant improvement in third molar angulation after premolar extraction compared to non-extraction treatment [17], whereas other studies refute such an association [14]. Some authors also suggest that factors other than premolar extraction providing more retromolar space could influence lower third molar angulation [14], including initial space conditions or mandibular growth. According to Richardson, the initial pretreatment angulation of the lower third molars may play a role in their subsequent eruption [29]. This is compatible with the results of the present study, where premolar extraction with subsequent space closure, the partial protraction of the first and second molar, and the potential increase of the retromolar space did not guarantee more uprighting of the third molar. Besides retromolar space, biological traits such as reduced mandibular length, vertical condylar growth, or retarded maturation of the third molar can influence its position.

In addition, no significant differences were found in the present study between patients having their first or second lower premolars extracted. A previous systematic review reported that the closer the extraction site is to the third molar, the more it influences its development and uprighting [21]. However, other authors also report similar trajectories for the third molars, irrespective of whether the first or second premolar was extracted [13, 16, 23, 30], indicating that treatment mechanics might play a greater role [30].

No difference in the posttreatment angulation between the right and left third molar was observed in this study. This makes sense since orthodontic fixed appliances were placed on all teeth of both jaws and similar bilateral mechanics were applied on both jaws. Opposite sides of the mandible are generally strongly correlated and are thought to be exposed to similar genetic and environmental factors [18, 41]. As rotational movements of the third molar bud usually occur, by close relationship with the second molar, it can be possible that depending on the second molars’ root morphology and position one side might start uprighting earlier than the other [33]. Previous studies have indicated that differences exist physiologically between the right and left side in terms of bite force [28] or tightness of the contact points between adjacent posterior teeth [45]. Nevertheless, it is questionable whether different environmental factors on each side alone could strongly influence the angulation of the third molars on either side.

There were some hints that premolar extraction treatment might influence the development/mineralization of the lower third molar, since extraction treatment was associated with increased odds of the lower right third molar being in a developmental stage where at least half of its root length was formed (Demirjian stages G–H; Table 5; [9]). This acceleratory phenomenon of premolar extractions on the eruption of third molars agrees with some authors [36] but not with others [23], who found no significant differences in the Demirjian classification between patients treated with or without premolar extractions and further studies are needed. Historical data from Björk’s longitudinal studies indicate that the mineralization stage of the third molars is closely associated with its eruption and late mineralization can be used to evaluate the risk of third molar impaction [37]. Therefore, if these findings are confirmed by future studies, late mineralization of third molars might be taken into account when deciding to whether to extract premolars among borderline cases, in order to potentially reduce the risk of third molar impaction.

Previous studies indicated that the angulation of the lower third molars improve after orthodontic treatment regardless of the treatment strategy [14, 34, 38], which indicates that this improvement might be attributed to the third molars’ physiological eruption path or to the growing processes of the mandible. The present study focused on the posttreatment OPGs of treated patients to gather information about the final angulation of the lower third molars and did not assess changes of angulation between the OPGs before and after orthodontic treatment. Therefore, the requirement of a mineralized third molar crown being present in the pretreatment OPG implies that considerably older patients would have to be selected, which might influence the measured angulation of the third molar.

Some variability was noticed in the repeated measurements, which indicated potential differences between either the two assessors or the same assessor between the different assessments (Supplementary Table 1). However, the average differences were very small and the reliability of OPGs for the measurement of third molar angulation has been proven [23, 46]. Although Tronje et al. [42] suggested that rotational panoramic radiography causes inbuilt distortion effects, they also stated that panoramic radiographic images can be considered reliable for geometric measurements in clinical practice. Akcam et al. [1] suggested that angular measurements on lateral cephalograms are less reliable, but others [35] noted that linear vertical measurements, ratio calculations, and angular measurements can be made on a panoramic radiograph with consistent accuracy [43].

A plethora of articles are available comparing the changes in third molar angulation between extraction and non-extraction groups. The parameters utilized in these studies, however, vary considerably. For example, the reference plane against which the angulation of the lower third molar is measured varies among the published research protocols: two studies used the occlusal plane which was defined by the occlusal surface of the posterior teeth [12, 34]. One [34] found no difference in the alteration of the lower third molar angulation between the extractions and non-extractions group, whereas the other [12] found not only that this difference was statistically significant but also that the available space increased significantly and the distance between the lower third molar and the occlusal plane decreased also significantly.

In as much, a variety of reference points has been used by various researchers to estimate the angulation of the third molar. Thus, the mandibular plane has been utilized as a reference plane in other studies [4, 31, 43] of which only Shashidhar et al. [31] found a statistically significant difference in the alteration of the angulation of the lower third molar by examining only cephalometric x‑rays, whereas the other two studies examined both cephalometric and panoramic x‑rays. Moreover, the Frankfurt plane was used as the reference plane by two studies [14, 39], which both examined panoramic x‑rays and found no difference in the angulation of the lower third molar before and after the orthodontic treatment between an extraction and non-extraction group. The use of the angulation of the third with the second molar was used only by Hartono et al. [16] who found no statistical difference before and after the orthodontic treatment in a group of patients with premolar extractions. Both the occlusal plane and the long axis of the second molar were used as references by others [2, 27]. All of them used a control group and panoramic x‑rays and only Durgesh et al. [11] found no statistically significant difference of the alteration of the angulation of the lower third molar between the extraction and non-extraction group.

A point that can be raised with regard to the calculation of the third molar angulation relative to the mandibular plane is that the remodeling of the lower border of the mandible could change the values that are to be measured. In our study, we evaluated the angle formed between the long axis of the third lower molar and the long axis of the second lower molar. According to Shashidhar et al. [31], this is not a stable measurement as the second molars can be tipped in a mesial/distal/lingual/buccal direction prior to the start of the treatment and then get corrected during orthodontic treatment. To avoid the implication of the second molar’s spatial orientation in investigating the third molar angulation, the lower third molar angulation was evaluated only in the posttreatment OPG.

The present study has also several limitations. For one, its retrospective character might subject it to confounding bias and selection bias relative to prospective and especially randomized studies [26]. In addition, space closure/retraction mechanics (including the direction from which the extraction spaces were closed) were not determined, although the third molar angulation/position is in the vast majority of cases not considered when choosing these mechanics. However, the fact that the first 120 cases that met the eligibility criteria were consecutively included might have minimized selection bias.

## Conclusions

Under the limitations of the present retrospective cross-sectional study, orthodontic treatment with bilateral extraction of premolars might be associated with small changes in the developmental stage of the lower third molars compared to patients treated without premolar extractions. However, it remains unclear if these effects can be generated to both left and right sides and whether they are influenced by the premolar extraction scheme, i.e., the first or the second premolar. It would be interesting to see future studies prospectively assessing the effect of extracting first or second premolars on the development of third molars according to the baseline posterior space availability and to the differential closure of extraction spaces from the anterior or the posterior side.

## Supplementary Information

Supplementary Table 1
