# Supplementary material for: Effect of orthodontic extraction of mandibular premolars on third molar angulation after treatment with fixed appliances: A cross-sectional study
Source: J Orofac Orthop. 2023 Mar 31;85(6):392–403. doi: 10.1007/s00056-023-00465-3 (PMC11496369; doi:10.1007/s00056-023-00465-3)
Supplement: Supplementary file 1 — Supplementary Table 1 [file 56_2023_465_MOESM1_ESM.pdf]

Supplementary Table 1. Agreement / reliability of repeated measurements.

Variable	Comparison	CCC (95% CI)	Average Difference (95% limits of agreement)
Angulation 48	Inter-examiner	0.93 (0.91, 0.96)	0.63 (-9.11, 10.37)
	Intra-examiner	0.98 (0.98, 0.99)	0.31 (-4.56, 5.18)
Angulation 38	Inter-examiner	0.94 (0.93, 0.97)	1.41 (-7.00, 9.82)
	Intra-examiner	0.99 (0.98, 0.99)	0.70 (-3.50, 4.91)

Abbreviations: CCC, concordance correlation coefficient; CI, confidence interval
